# Preliminary evaluation of the larvicidal activity of extracts and fractions from *Ocotea nutans* (Nees) Mez against *Aedes aegypti*

**DOI:** 10.1590/0037-8682-0576-2020

**Published:** 2021-02-26

**Authors:** Fernando Cesar Martins Betim, Camila Freitas de Oliveira, Deise Prehs Montrucchio, Obdulio Gomes Miguel, Marilis Dallarmi Miguel, Juliana Bello Baron Maurer, Josiane de Fátima Gaspari Dias

**Affiliations:** 1 Universidade Federal do Paraná, Programa de Pós-Graduação em Ciências Farmacêuticas, Curitiba, PR, Brasil.; 2 Universidade Estadual do Centro Oeste, Departamento de Farmácia, Guarapuava, PR, Brasil.; 3 Universidade Federal do Paraná, Departamento de Bioquímica e Biologia Molecular, Curitiba, PR, Brasil.

**Keywords:** Ocotea nutans, Larvicidal potential, Aedes aegypti, Brazilian plant

## Abstract

**INTRODUCTION:**

*Aedes aegypti* is the main vector of dengue and yellow fever. Recently, the use of plant-sourced larvicides has gained momentum.

**METHODS:**

The hydroethanolic extracts and fractions of*Ocotea nutans*leaves and stems were bioassayed to determine the larvicidal efficacy of these samples.

**RESULTS:**

S-HEX (hexane fraction from the crude stem extract) demonstrated high potential for controlling third-stage larvae, with an LC_50_ of 14.14 µg.mL^-1^ (concentration required to inhibit 50% of the treated larvae).

**CONCLUSIONS:**

Extracts from *O. nutans* were effective against third-stage larvae of*A. aegypti*after 24 h of exposure.

The*Aedes aegypti*Linnaeus (Diptera: Culicidae) mosquito is a vector for arboviruses, which cause diseases such as chikungunya fever, yellow fever, dengue, and Zika and therefore, constitute a public health problem^1^. Recently, the number of cases of dengue and dengue hemorrhagic fever has increased sharply, affecting developing countries worldwide. Studies have reported the failure of traditional control methods due to insecticide resistance developed by the vector, its continuous dissemination, and the occurrence of epidemics^2^. 

Panneerselvam et al.^3^ and Seetharaman et al.^4^ have reported that the overuse of conventional synthetic pesticides and larvicidal agents has influenced the emergence of resistant mosquito populations. The Brazilian flora, which is both extensive and diverse, is a natural asset possessing immense potential for secondary plant compounds, many of which can be utilized in the manufacture of agrochemicals. The search for novel compounds with larvicidal and insecticidal activities has gained further significance as the region is currently experiencing a dramatic increase in the incidence of insect-transmitted diseases. 

Plant-based insecticides have been evaluated for the control of vectors related to human diseases. The use of botanical insecticides could induce mortality during different stages, retard growth, render adult insects infertile, and decrease the viability of insect eggs^5^.

*Ocotea nutans* (Ness) Mez, commonly known as canelinha or canela in Brazil, is a tree that reaches 10-30 m in height. This native Brazilian species inhabits mixed ombrophilus forests and is found in the states of Paraná, Sao Paulo, Espírito Santo, Minas Gerais, and Bahia^6^. The essential oil of *O. nutans* contains bicyclogermacrene (11.41%), germacrene-D (4.89%), bisabolol-11-ol (3.73%), and spathulenol (3.71%) as the major compounds; it shows toxicity against *A. aegypti* and controls third-stage larvae^7^, with an LC_50_ of 250 µg.mL^-1^.

Considering the larvicidal potential of *O. nutans* essential oil, in this study, we aimed to evaluate the effects of the extracts and fractions obtained from *O. nutans* leaves and stems on *A.aegypti* larvae under laboratory conditions.

The leaves and stems of *O. nutans* were collected from the city of Curitiba, Paraná, Brazil (25°26'55"S, 49°14'22"W). Plant identification was performed by the forest engineer, Marcelo Leandro Brotto, from the Botanical Garden of Curitiba Herbarium,and the plant was compared with a voucher specimen deposited under number 56552 at the Herbarium of the Federal University of Paraná. 

This study was authorized by SisGen, a legislative and deliberative body under the Ministry of Environment of Brazil, under the number A0EB51A, which grants permission to evaluate the bioactivities of extracts derived from Brazilian plants.

The leaves and stems were dried at 50 °C. A crude ethanolic extract was prepared with 96 ºGL ethanol (1:10; *w/v*) in a Soxhlet apparatus under continuous reflux for 6 h at 80 ºC. Fractions were obtained by employing the liquid-liquid partitioning method of using solvents in increasing order of polarity (*n*-hexane, chloroform, and ethyl acetate) in a modified Soxhlet apparatus^8^. This technique was separately performed for leaves and stems to obtain crude ethanolic extracts from leaves (L-CEE) and stems (S-CEE).

After fractioning, four organic fractions were obtained from each crude extract: hexane fraction (L-HEX), chloroform fraction (L-CHL), ethyl acetate fraction (L-ETH), and a residual fraction (L-RES) from L-CEE; additionally, a hexane fraction (S-HEX), chloroform fraction (S-CHL), ethyl acetate fraction (S-ETH), and a residual fraction (S-RES) were obtained from S-CEE. Next, the extracts and fractions were vacuum filtered and concentrated in a rotary evaporator under reduced pressure at 40 °C.

The following methodology was adapted from Betim et al.^7^ and Garcez et al.^9^. The eggs of *A. aegypti* (Rockefeller strain provided by the Oswaldo Cruz Foundation) were reared under laboratory conditions (27±3 °C, relative humidity of 80%, and incubated in a Bio-Oxygen Demand incubator) by feeding with fish feed (Aldon basic, MEP 200 complex) from hatching until the third stage of larval development. The samples were diluted in 0.5% dimethyl sulfoxide (DMSO) and then dissolved in dechlorinated water to obtain the desired concentration (1000, 100, and 10 µg.mL^-1^). After hatching, 10 larvae were treated with the controls (water and DMSO) as well as with extracts and fractions for 24h. Next, living and dead larvae were counted in triplicate for each treatment, reaching a total of 30 larvae for each sample dose.

The probit method was used to determine the lethal concentration (LC_50_ and LC_90_) values as well as the corresponding 95% confidence intervals; the chi-square test was used for the *A. aegypti* assay. All statistical analyses were performed using IBM SPSS Statistics version 20.0 (IBM Corp., Armonk, NY, USA).

Based on our findings, it was confirmed that the extract and fractions obtained from *O. nutans* induced mortality in *A. aegypti* larvae (Table 1). Pronounced larvicidal effects were observed with the *n-*hexane and chloroform fractions of leaves and stems. S-HEX demonstrated high potential for controlling third-stage larvae, with an LC_50_ of 14.14 µg.mL^-1^ (required to inhibit 50% of treated larvae). L-HEX, with an LC_50_ of 111.32 µg.mL^-1^, and L-CHL, with an LC_50_ of 171.41 µg.mL^-1^, revealed the greatest potential for controlling third-stage larvae. Additionally, S-CHL showed larvicidal potential with an LC_50_ of 441.4 µg.mL^-1^. Other extracts and fractions induced low or no mortality in third-stage larvae. 

The larval mortality profile, with regard to the concentrations of extracts and fractions of *O. nutans*, is shown in Table 1. The mortality profile was accentuated for S-HEX, L-HEX, S-CHL, and L-CHL, mainly at concentrations of 1000 µg.mL^-1^.

**TABLE 1:** Induction of mortality in *A. aegypti* larvae by extracts and fractions from *Ocotea nutans.*

Sample	Concentration	Mortality (%) ± SD	LC_50_ (µg.mL^-1^)	LC_90_ (µg.mL^-1^)	x²	(df)
	(µg.mL^-1^)		(LCL - UCL)	(LCL - UCL)		
L-CEE	10	3.33 ± 0.57	> 1000	> 1000	n.d.	n.d.
	100	33.33 ± 0.57				
	1000	40 ± 0.00				
L-HEX	10	16.66 ± 0.57	111.32	> 1000	2.38	1 (n.s.)
	100	53.33 ± 1.15	(48.02 - 263.96)			
	1000	76.66 ± 0.57				
L-CHL	10	23.33 ± 0.57	171.41	> 1000	1.91	1 (n.s.)
	100	50 ± 1.00	(47.7 - 1129.1)			
	1000	63.33 ± 0.57				
L-ETH	10	0.0 ± 0.00	> 1000	> 1000	n.d.	n.d.
	100	0.0 ± 0.00				
	1000	10 ± 0.00				
L-RES	10	0.0 ± 0.00	> 1000	> 1000	n.d.	n.d.
	100	0.0 ± 0.00				
	1000	1.0 ± 1.00				
S-CEE	10	16.66 ± 0.57	> 1000	> 1000	n.d.	n.d.
	100	23.33 ± 0.57				
	1000	50± 1.00				
S-HEX	10	46.66 ± 0.57	14.14	207,55	2.20	1 (n.s.)
	100	76.66 ± 0.57	(4.3 - 23.18)	(93,6 - 1120,9)		
	1000	100.00 ± 0.00				
S-CHL	10	6.66 ± 0.57	441.4	> 1000	3.76	1 (n.s.)
	100	26.66 ± 1.52	(209.3 - 2309.6)			
	1000	63.33 ± 0.57				
S-ETH	10	0.0 ± 0.00	> 1000	> 1000	n.d.	n.d.
	100	13.33 ± 1.15				
	1000	23.33 ± 1.15				
S-RES	10	0.0 ± 0.00	> 1000	> 1000	n.d.	n.d.
	100	0.0 ± 0.00				
	1000	0.0 ± 0.00				

Legend: Leaves crude ethanolic extract (L-CEE), leaves hexane fraction (L-HEX), leaves chloroform fraction (L-CHL), leaves ethyl acetate fraction (L-ETH), leaves residual fractions (L-RES). Stem crude ethanolic extract (S-CEE), stem hexane fraction (S-HEX), stem chloroform fraction (S-CHL), stem ethyl acetate fraction (s-ETH), stem residual fractions (S-RES). Notes: no mortality was observed in the negative controls; positive control killed 100% larvae in*A. aegypti*; (LC_50_) lethal concentration that kills 50% of the exposed organisms; (LC_90_) lethal concentration that kills 90% of the exposed organisms; (UCL) 95% upper confidence limit; (LCL) 95% lower conﬁdence limit; x^2^ = chi-square statistics; df = degrees of freedom; (n.d.) not defined; (n.s.) not significant (p<0.05).

The Brazilian flora is rich in *Ocotea* plants, and extracts and fractions from several different species have already been evaluated for their larvicidal potential. Garcez et al.^9^ examined the potential larvicidal properties of ethanolic extracts obtained from the leaves, fruits, and trunk bark of *O. minarum*(Nees & C. Mart.) Mez against *A. aegypti,* reporting no mortality (LC_50_>1000 µg.mL^-1^ *).* The ethanolic extract was derived from the trunk of *O. suaveolens*(Meisn) Benth. & Hook.f. ex Hieron. was evaluated against *A. aegypti* larvae, with no mortality recorded (LC_50_> 1000 µg.mL^-1^)^9^. Notably, the ethanolic trunk bark extract from *O. velloziana*(Meisn.) Mez presented superior potential against*A. aegypti* larvae (LC_50_= 213,70 µg.mL^-1^)^9^.

The low LC_50_ of L-HEX and S-HEX shows that mortality was induced by this fraction because of the presence of a rich mixture of apolar compounds such as lignans, terpenes, and derivates. In the literature, extracts produced with hexane indicate the possibility of apolar compound extraction. Narciso et al.^10^examined the larvicidal properties of lignin extracted from the hexane fractions of stems from *O. cymbarum* Kunth and reported high mortality against *A. aegypti* larvae. Betim^7^ et al. examined essential oils (terpenoid-based constituents) from *O. nutans*, and this mixture could control third-stage larvae, with an LC_50_ of 250 µg.mL^-1^. Terpenes and derivatives have larvicidal properties, and it is suggested that their liposoluble characteristics have a strong influence on the mortality of larvae^11^.

Mortality was induced by the L-CHL and S-CHL fractions due to the presence of a rich mixture of alkaloids and nitrogenated derivatives. Extracts produced with chloroform indicated the possibility of nitrogenated compound extraction (e.g., alkaloids), and these metabolites have great potential in larvicidal activities. Dicentrin^9^, an alkaloid isolated from *O. velloziana and tested* in the third stage of *A. aegypti* larvae, had an LC_50_ of 30 µg.mL^-1^. The alkaloids showed larvicidal activity and had a mode of action similar to that of natural pyrethrin insecticides (basis for synthetic insecticides, e.g., pyrethroids). The phytolarvicidal action of alkaloids also has a similar action to the carbamate and organophosphate insecticides^12^. The genus *Ocotea* has a high possibility of containing the secondary metabolites derived from terpenoids and alkaloids, as described in the literature.

Notably, virus transmission by *A. aegypti* can be prevented or reduced through environmental management and by using synthetic insecticides belonging to pyrethroids or organophosphates, including temephos^13^, to minimize the spread of mosquitoes and human contact. However, the frequent use of synthetic pesticides to control the *A. aegypti* population can result in environmental and/or human contamination, along with the emergence of resistant insects^14^.

For vector control in public health, chemical control utilizing insecticides of organic or inorganic origin is one of the most frequently adopted strategies for sustainable, integrated management. However, its continued use has increased the appearance of resistant populations, leading to challenges in vector control. Moreover, resistance has been detected for all classes of insecticides, directly and profoundly affecting the re-emergence of vector-borne diseases. This is because despite important advances in the development of alternative methods, chemical insecticides remain an important tool in programs undertaking integrated control^15^.

Notably, the frequent use of these insecticides has resulted in phytotoxicity, human poisoning, and emergence of resistant insects^8^ ^,^ ^9^. Hence, researchers are attempting to develop alternative strategies to control *A. aegypti* proliferation, including the use of phytolarvicides composed of plant extracts or compounds^5^ ^,^ ^7^ ^,^ ^8^.

Plant-based compounds are already known to exhibit larvicidal properties, which could be useful for the development of eco-friendly larvicides^7^ ^,^ ^14^.

Under laboratory conditions, the hexane and chloroform fractions of the leaves and stems of *O. nutans* demonstrated larvicidal effects against *A. aegypti*. These results present an opportunity to replace synthetic pesticides with natural products in vector control programs for yellow fever, dengue, and, more recently, chikungunya. Moreover, the efficacy of the hexane stem fraction is promising. The potential of extracts derived from these plants as larvicides against *A. aegypti* represents an abundant and accessible alternative in southern Brazil, where increasing infestations and dengue cases have been reported in the last decade. 

Furthermore, these extracts presented an alternative to the synthetic products recommended by the Ministry of Health for the control of dengue. Superior results can be obtained with additional studies to evaluate the larvicidal activity of pure compounds isolated from these plants.
